# Soil microbial biomass and enzyme data after six years of cover crop and compost treatments in organic vegetable production

**DOI:** 10.1016/j.dib.2018.09.013

**Published:** 2018-09-12

**Authors:** Eric B. Brennan, Veronica Acosta-Martinez

**Affiliations:** United States Department of Agriculture, Agricultural Research Service, USA

## Abstract

Cover crops and compost are organic matter inputs that can impact soil health in tillage-intensive, high-input, organic vegetable production systems in the central coast region of California. Data are presented on soil microbial biomass (carbon and nitrogen) and soil enzymes (β-glucosidase, β-glucosaminidase, alkaline phosphatase, aspartase and L-asparaginase and dehydrogenase) from a relatively long-term organic systems experiment in Salinas, California that was focused on lettuce and broccoli production and included eight different certified organic systems. These systems differed in compost inputs, cover cropping frequency, cover crop type, and cover cropping seeding rate. The compost was made from urban yard waste, and the cover crops included rye, a legume-rye mixture, and a mustard mixture planted at two seeding rates (standard rate 1× versus high rate 3×). There were three legume-rye 3× systems that differed in compost inputs (0 versus 15 Mg ha^-1^ year^-1^ and cover cropping frequency (every winter versus every fourth winter). The data in this article support and augment information presented in the research articles “Cover cropping frequency is the main driver of soil microbial changes during six years of organic vegetable production” (Brennan and Acosta-Martinez, 2017) and “Cover crops and compost influence soil enzymes during 6 years of tillage-intensive, organic vegetable production” (Brennan and Acosta-Martinez, 2018).

**Specifications table**

**Table:** 

Subject area	*Agriculture*
More specific subject area	*Soil microbiology, nutrient management, vegetable production, long-term organic systems research*
Type of data	*Tables, figures*
How data was acquired	The activities of β-glucosidase, β-glucosaminidase, alkaline phosphatase, and dehydrogenase were determined by incubating soil in appropriate substrates, extracting the reaction product, and colorimetric determination of the reaction product using a spectrophotometer (Beckman Coulter DU640, Brea, CA). The activities of aspartase, L-asparaginase were determined by steam distillation with a Foss Kjeltec 2200 Auto Distillation Unit (Foss North America, Eden Prairie, MN) to collect the product of reaction into the distillate (release of amide and converted into ammonia/ammonium) and titration with a Mettler Toledo DL 50 titrator (Mettler-Toledo Inc., Columbus, OH). Microbial biomass (C and N) were measured with the chloroform fumigation extraction method.
Data format	*Raw, descriptive and inferential*
Experimental factors	*Cover cropping frequency, cover crop type, cover crop seeding rate, compost application rate.*
Experimental features	*The soil was collected in October, 2003 (Time 0, before the treatments began) and October, 2009 (after 6 years the experimental treatments) from 6 to 8 core samples per plot from a depth of 0 to 6.5 cm. The cores were mixed to produce a composite sample for each experimental plot. The soil was stored frozen at -25 C prior to determination of soil enzyme activities and microbial biomass that were conducted in 2009 and 2010.*
Data source location	*Salinas, California, United States of America. lat. 36.622658, long. -121.549172, elevation 37 m above sea level.*
Data accessibility	*The data on soil enzymes and microbial biomass (carbon and nitrogen) are in the tables and figures in this article. The bacterial sequence data summarized in our related article from 2017 is available in the public repository National Center for Biotechnology Information under Bioproject PRJNA344674*https://www.ncbi.nlm.nih.gov/bioproject/PRJNA344674*with accession numbers: SRR4300068, SRR4300077, SRR4300078, SRR4300079, SRR4300080, SRR4300081, SRR4300086, SRR4300087, SRR4300089, SRR4300094, SRR4300095, SRR4300138, SRR4300139, SRR4300140, SRR4300145, SRR4300149, SRR4300150, SRR4300151, SRR4300152, SRR4300153, SRR4300154, SRR4300155, SRR4300156, SRR4300242, SRR4300243, SRR4300244, SRR4300264, SRR4300272, SRR4300284, SRR4300294.*
Related research article	– Brennan, E.B. and V. Acosta-Martinez, 2018. Cover crops and compost influence soil enzymes during 6 years of tillage-intensive, organic vegetable production. Soil Sci. Soc. Am. J. 82. *In Press*. – Brennan, E.B. and V. Acosta-Martinez, 2017. Cover cropping frequency is the main driver of soil microbial changes during six years of organic vegetable production. Soil Biol. Biochem. 109:188–204.

**Value of the data**

• The data is from the first six years of the longest running organic systems study in the U.S. that is focused on high-value, high-input, tillage-intensive, organic vegetable production. This is the most important region of the U.S. for high-value, cool season vegetable production.
• Soil enzymes and soil microbial biomass (carbon and nitrogen) are sensitive, early indicators of changes in soil health, but are not well-understood in tillage-intensive production systems. This data could be valuable in future meta-analyses that seek to understand the complex effects of compost and cover crops in vegetable systems. The data augment our related publications that only included data from 5 of the 8 systems in the long-term study.
• The data may serve as a benchmark for future studies of soil enzymes and microbial biomass in a loamy sand soil in California and other regions with a Mediterranean climate.
• This data may be useful to develop more sustainable organic and conventional vegetable systems in many regions of the world. For example, it may serve as a benchmark in the development of reduced tillage systems for vegetable production in this region and elsewhere.
• This data enables others to independently evaluate or extend the statistical analyses presented in the related articles. This may be useful to help researchers and students to understand the statistical analysis approach that was focused on point and interval estimates in the related articles. This statistical analysis approach used the software known as the Exploratory Software for Confidence Intervals (ESCI) that is freely available online.

## 1 Data

**Fig. 1:**
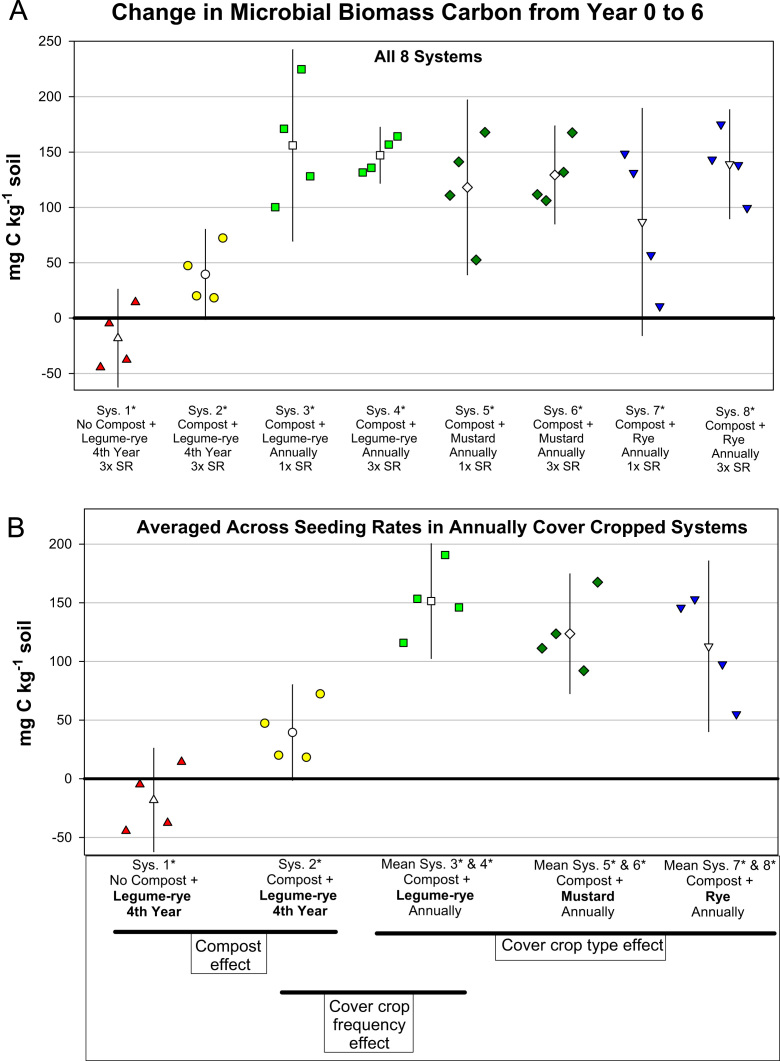
Change in microbial biomass carbon from year 0 to year 6 in all eight systems (A) and averaged across the 1× and 3× seeding rates (SR) in the annually cover cropped systems (B) in the Salinas Organic Cropping Systems experiment in Salinas, California. The systems differed in compost additions (none versus 15.2 Mg ha^-1^ annually), cover crop type (legume-rye, mustard, or rye), cover cropping frequency (every 4th winter versus annually) and cover crop seeding rate (1×= standard rate versus 3×= high rate); see Table 1 for more seeding rate details. Symbols are raw data in order of replicates 1 to 4 with mean and 95% confidence interval (CI) in the center of each data cluster. The horizontal lines below the system labels on the x-axis in plot B show the systems that can be compared to evaluate the effects of compost, cover crop frequency, and cover crop type. Plot B that is averaged across both seeding rates in the annually cover cropped systems is similar and complementary to Fig. 2B in the related article [1] that included only 5 systems (1*, 2*, 4*, 5*, 7*); see Table 1 in the present article for more details.

**Fig. 2:**
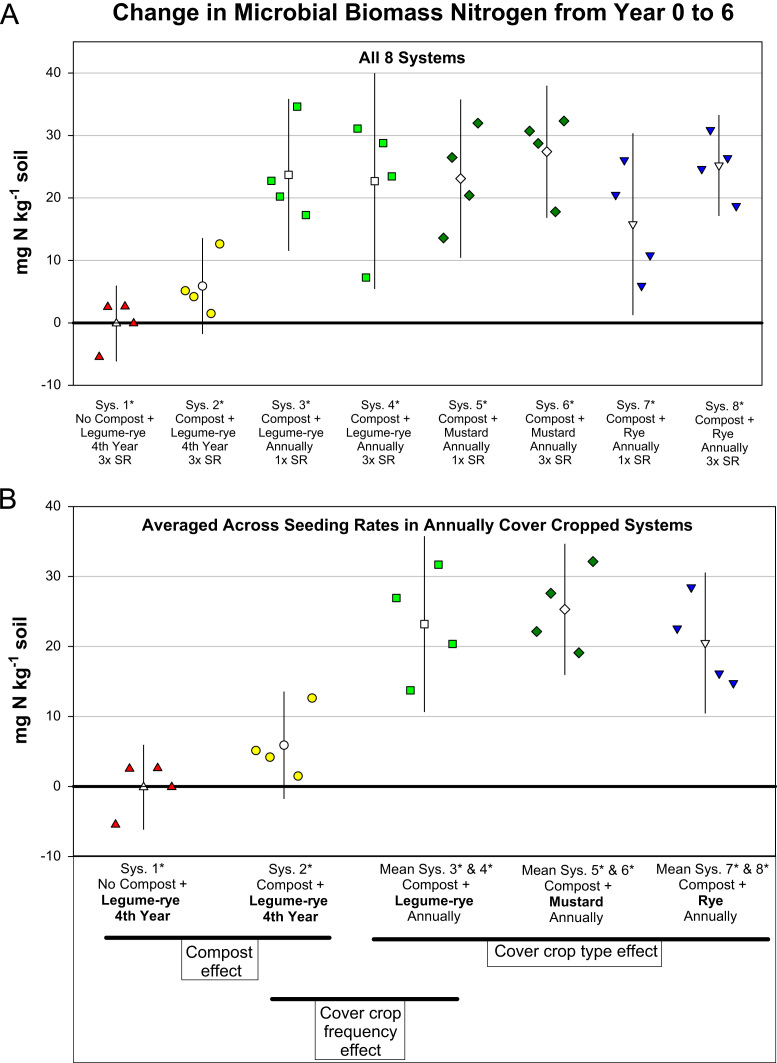
Change in microbial biomass nitrogen from year 0 to year 6 in all eight systems (A) and averaged across the 1× and 3× seeding rates (SR) in the annually cover cropped systems (B) in the Salinas Organic Cropping Systems experiment in Salinas, California. The systems differed in compost additions (none versus 15.2 Mg ha^-1^ annually), cover crop type (legume-rye, mustard, or rye), cover cropping frequency (every 4th winter versus annually) and cover crop seeding rate (1×= standard rate versus 3×= high rate); see Table 1 for more seeding rate details. Symbols are raw data in order of replicates 1 to 4 with mean and 95% confidence interval (CI) in the center of each data cluster. The horizontal lines below the system labels on the x-axis in plot B show the systems that can be compared to evaluate the effects of compost, cover crop frequency, and cover crop type. Plot B that is averaged across both seeding rates in the annually cover cropped systems is similar and complementary to Fig. 3B in the related article [1] included only 5 systems (1*, 2*, 4*, 5*, 7*); see Table 1 in the present article for more details.

**Fig. 3:**
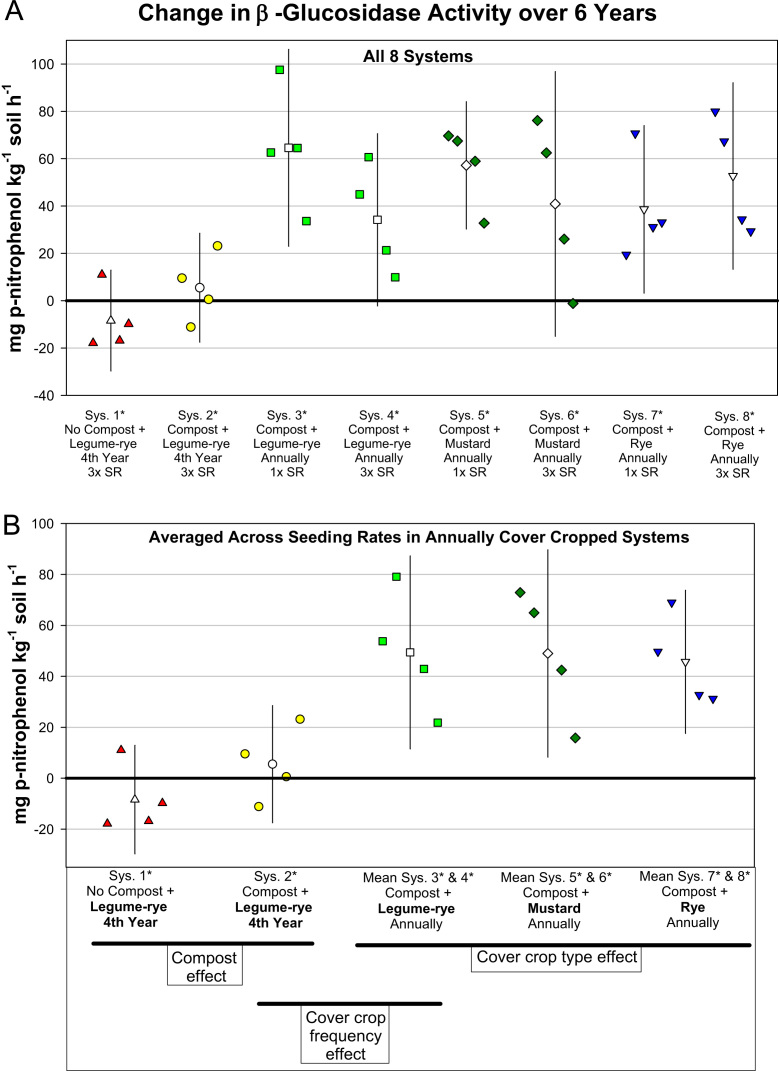
Change in β-glucosidase activity from year 0 to year 6 in all eight systems (A) and averaged across the 1× and 3× seeding rates (SR) in the annually cover cropped systems (B) in the Salinas Organic Cropping Systems experiment in Salinas, California. The systems differed in compost additions (none versus 15.2 Mg ha^-1^ annually), cover crop type (legume-rye, mustard, or rye), cover cropping frequency (every 4th winter versus annually) and cover crop seeding rate (1×= standard rate versus 3×= high rate); see Table 1 for more seeding rate details. Symbols are raw data in order of replicates 1 to 4 with mean and 95% confidence interval (CI) in the center of each data cluster. The horizontal lines below the system labels on the x-axis in plot B show the systems that can be compared to evaluate the effects of compost, cover crop frequency, and cover crop type. Plot B that is averaged across both seeding rates in the annually cover cropped systems is similar and complementary to Fig. 1B in the related article [2] that included only 5 systems (1*, 2*, 4*, 5*, 7*); see Table 1 in the present article for more details.

**Fig. 4:**
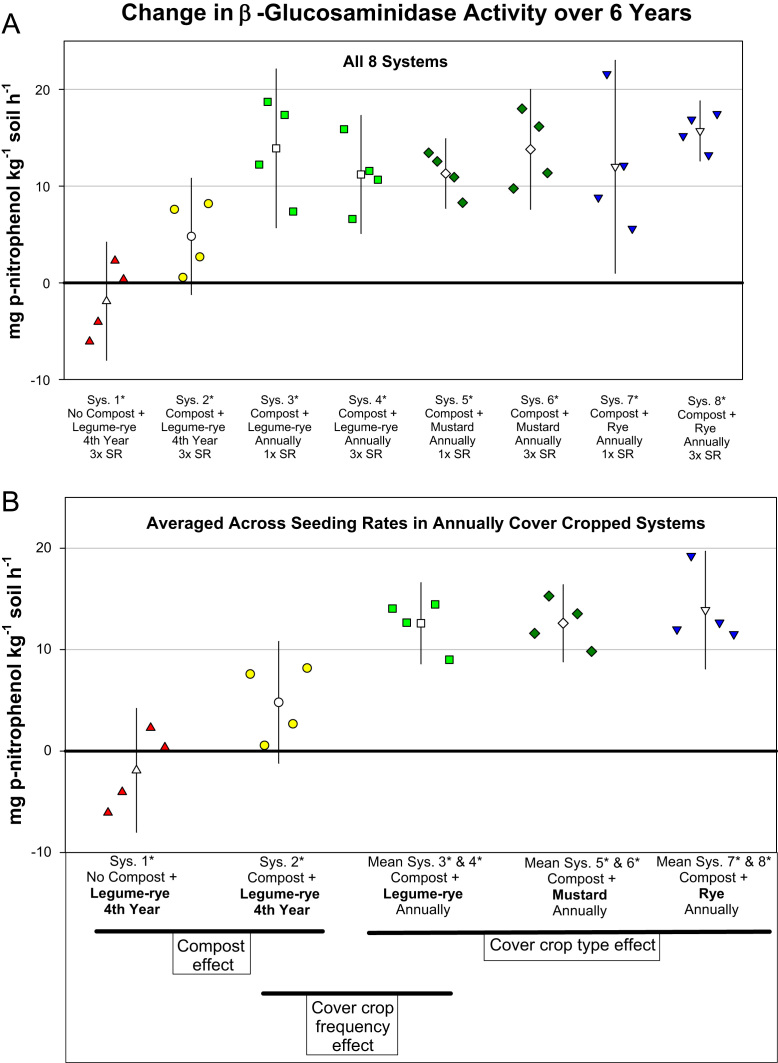
Change in β-glucosaminidase activity from year 0 to year 6 in all eight systems (A) and averaged across the 1× and 3× seeding rates (SR) in the annually cover cropped systems (B) in the Salinas Organic Cropping Systems experiment in Salinas, California. The systems differed in compost additions (none versus 15.2 Mg ha^-1^ annually), cover crop type (legume-rye, mustard, or rye), cover cropping frequency (every 4th winter versus annually) and cover crop seeding rate (1×= standard rate versus 3×= high rate); see Table 1 for more seeding rate details. Symbols are raw data in order of replicates 1 to 4 with mean and 95% confidence interval (CI) in the center of each data cluster. The horizontal lines below the system labels on the x-axis in plot B show the systems that can be compared to evaluate the effects of compost, cover crop frequency, and cover crop type. Plot B that is averaged across both seeding rates in the annually cover cropped systems is similar and complementary to Fig. 2B in the related article [2] that included only 5 systems (1*, 2*, 4*, 5*, 7*); see Table 1 in the present article for more details.

**Fig. 5:**
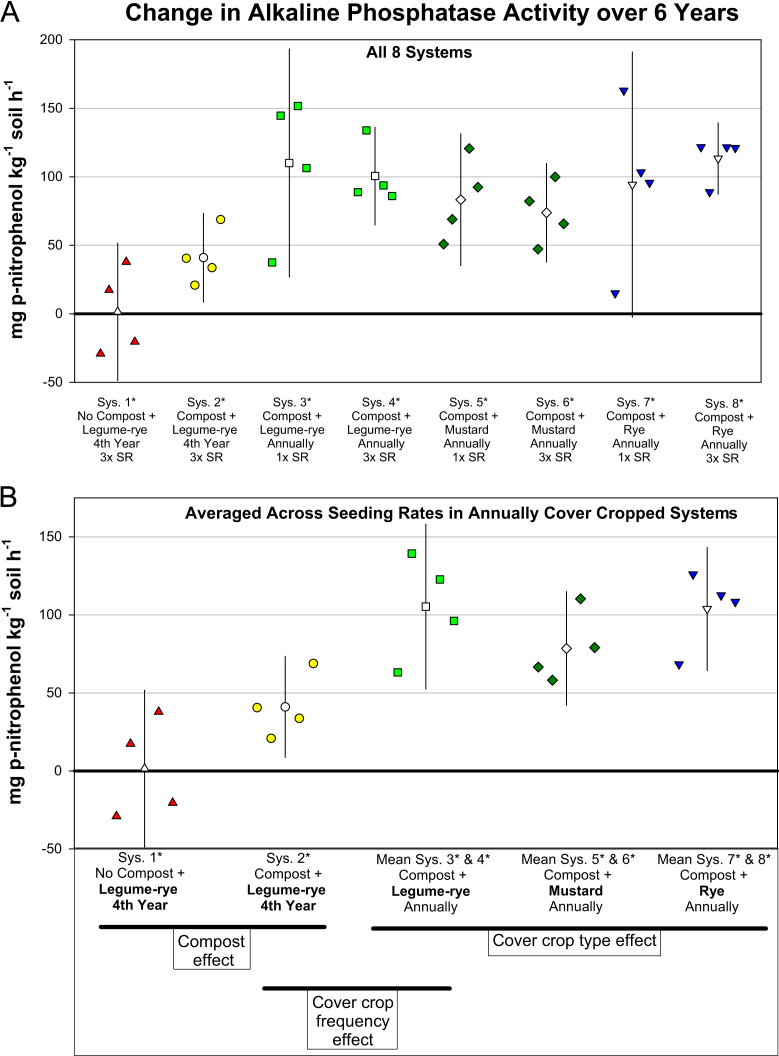
Change in alkaline phosphatase activity from year 0 to year 6 in all eight systems (A) and averaged across the 1× and 3× seeding rates (SR) in the annually cover cropped systems (B) in the Salinas Organic Cropping Systems experiment in Salinas, California. The systems differed in compost additions (none versus 15.2 Mg ha^-1^ annually), cover crop type (legume-rye, mustard, or rye), cover cropping frequency (every 4th winter versus annually) and cover crop seeding rate (1×= standard rate versus 3×= high rate); see Table 1 for more seeding rate details. Symbols are raw data in order of replicates 1 to 4 with mean and 95% confidence interval (CI) in the center of each data cluster. The horizontal lines below the system labels on the x-axis in plot B show the systems that can be compared to evaluate the effects of compost, cover crop frequency, and cover crop type. Plot B that is averaged across both seeding rates in the annually cover cropped systems is similar and complementary to Fig. 3B in the related article [2] that included only 5 systems (1*, 2*, 4*, 5*, 7*); see Table 1 in the present article for more details.

**Fig. 6:**
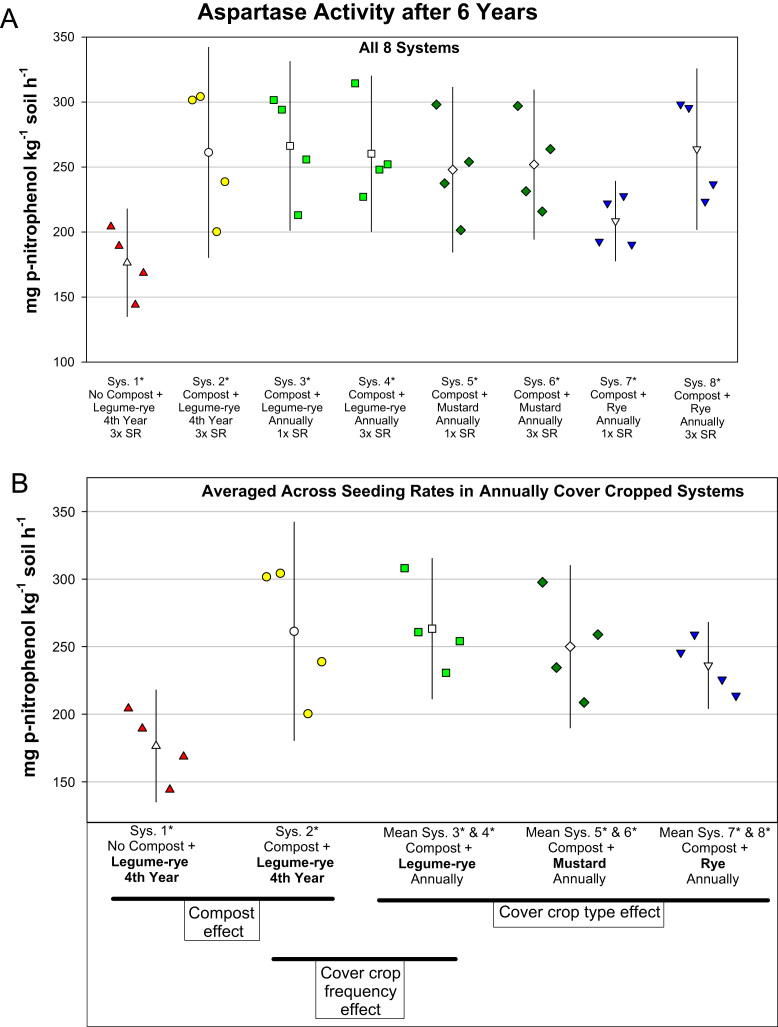
Aspartase activity after 6 years in all eight systems (A) and averaged across the 1× and 3× seeding rates (SR) in the annually cover cropped systems (B) in the Salinas Organic Cropping Systems experiment in Salinas, California. The systems differed in compost additions (none versus 15.2 Mg ha^-1^ annually), cover crop type (legume-rye, mustard, or rye), cover cropping frequency (every 4th winter versus annually) and cover crop seeding rate (1×= standard rate versus 3×= high rate); see Table 1 for more seeding rate details. Symbols are raw data in order of replicates 1 to 4 with mean and 95% confidence interval (CI) in the center of each data cluster. The horizontal lines below the system labels on the x-axis in plot B show the systems that can be compared to evaluate the effects of compost, cover crop frequency, and cover crop type. Plot B that is averaged across both seeding rates in the annually cover cropped systems is similar and complementary to Fig. 4A in the related article [2] that included only 5 systems (1*, 2*, 4*, 5*, 7*); see Table 1 in the present article for more details.

**Fig. 7:**
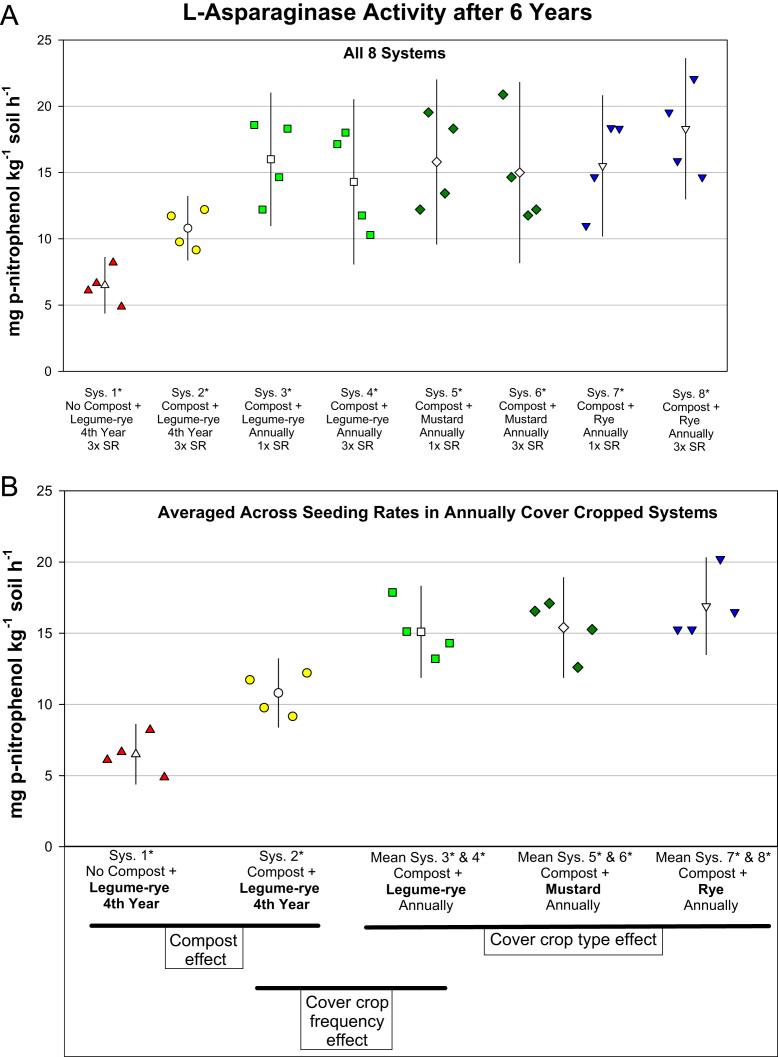
L-Asparaginase activity after 6 years in all eight systems (A) and averaged across the 1× and 3× seeding rates (SR) in the annually cover cropped systems (B) in the Salinas Organic Cropping Systems experiment in Salinas, California. The systems differed in compost additions (none versus 15.2 Mg ha^-1^ annually), cover crop type (legume-rye, mustard, or rye), cover cropping frequency (every 4th winter versus annually) and cover crop seeding rate (1×= standard rate versus 3×= high rate); see Table 1 for more seeding rate details. Symbols are raw data in order of replicates 1 to 4 with mean and 95% confidence interval (CI) in the center of each data cluster. The horizontal lines below the system labels on the x-axis in plot B show the systems that can be compared to evaluate the effects of compost, cover crop frequency, and cover crop type. Plot B that is averaged across both seeding rates in the annually cover cropped systems is similar and complementary to Fig. 4B in the related article [2] that included only 5 systems (1*, 2*, 4*, 5*, 7*); see Table 1 in the present article for more details.

**Fig. 8:**
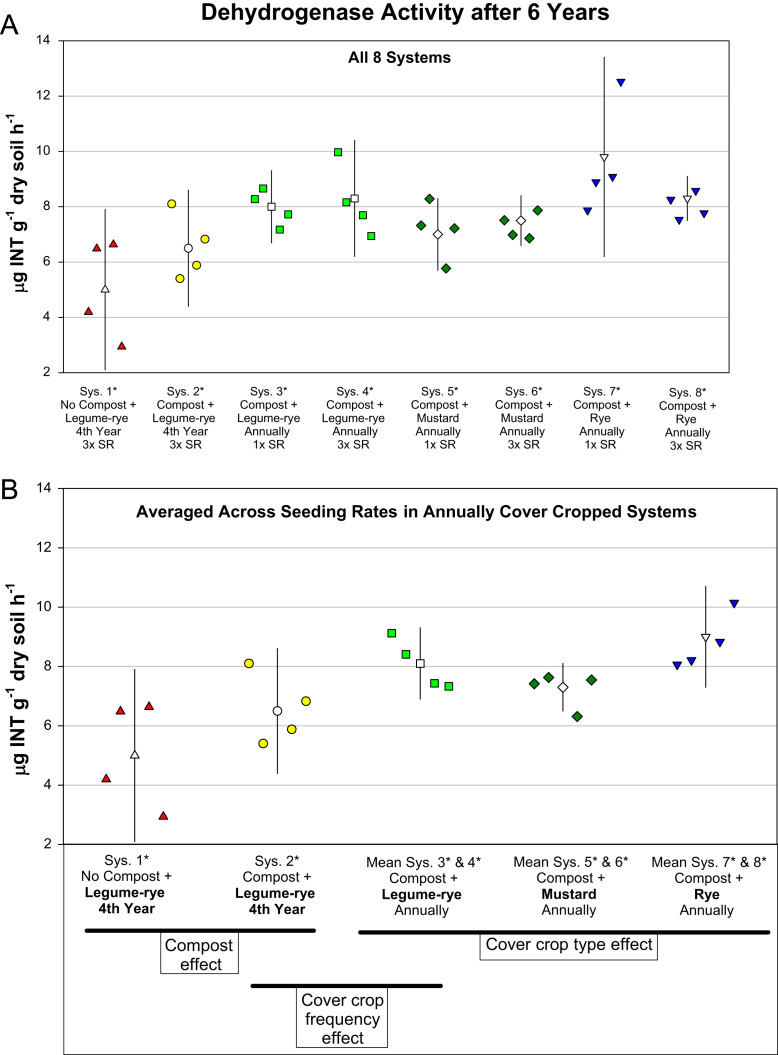
Dehydrogenase activity after 6 years in all eight systems (A) and averaged across the 1× and 3× seeding rates (SR) in the annually cover cropped systems (B) in the Salinas Organic Cropping Systems experiment in Salinas, California. The systems differed in compost additions (none versus 15.2 Mg ha^-1^ annually), cover crop type (legume-rye, mustard, or rye), cover cropping frequency (every 4th winter versus annually) and cover crop seeding rate (1×= standard rate versus 3×= high rate); see Table 1 for more seeding rate details. Symbols are raw data in order of replicates 1 to 4 with mean and 95% confidence interval (CI) in the center of each data cluster. The horizontal lines below the system labels on the x-axis in plot B show the systems that can be compared to evaluate the effects of compost, cover crop frequency, and cover crop type. Plot B that is averaged across both seeding rates in the annually cover cropped systems is similar and complementary to Fig. 4C in the related article [2] that included only 5 systems (1*, 2*, 4*, 5*, 7*); see Table 1 in the present article for more details.

This article includes the raw data, descriptive data (means) and inferential statistics (95% confidence intervals) on the effects of compost and cover cropping on changes in microbial biomass carbon, microbial biomass nitrogen, and soil enzymes activities over a 6 years period in the Salinas Organic Cropping Systems (SOCS) experiment (Table 1, Table 2, Fig. 1, Fig. 2, Fig. 3, Fig. 4, Fig. 5, Fig. 6, Fig. 7, Fig. 8). This important long-term study is located at the USDA-ARS (United States Department of Agriculture – Agricultural Research Service) organic research farm in Salinas, California and is approximately 24 km inland from Monterey Bay in a region commonly referred to as the ‘Salad Bowl of America’. This ongoing systems study was designed to provide information on the impact of yard waste compost and cover crops (type, frequency, and seeding rate) on a variety of aspects (ex., soil health, yields, weeds) of vegetable production.

**Table 1:** Descriptions of systems in the Salinas Organic Cropping Systems experiment in Salinas California.

System ID used in this *Data in Brief* article	System ID in SBB & SSSJA articles^a^		Cover crop			Compost input^e^ (Mg ha^-1^ 6 yr^-1^)	Total organic matter input^f^ (i.e., Cover crop + compost) (Mg ha^-1^ 6 yr^-1^)
			Type^b^	Frequency^c^	Seeding rate^d^		
1*		1	Legume-rye	4^th^ Winter	3×	0	7.4
2*		2	Legume-rye	4^th^ Winter	3×	91.2	99.2
3*			Legume-rye	Every Winter	1×	91.2	136.8
4*		3	Legume-rye	Every Winter	3×	91.2	136.8
5*		4	Mustard	Every Winter	1×	91.2	122.1
6*			Mustard	Every Winter	3×	91.2	123.6
7*		5	Rye	Every Winter	1×	91.2	134.3
8*			Rye	Every Winter	3×	91.2	135.2

a System ID code used in the related articles on microbial biomass in Soil Biology and Biochemistry (SBB) [1], and the Soil Science Society of America Journal (SSSJA) [2].

b By seed weight, the legume-rye mixture included 10% Rye (‘Merced’ *Secale cereale* L.), 35% Faba bean, (*Vicia faba* L.; small-seeded type known as ‘bell bean’), 25% Pea, ‘Magnus’ *Pisum sativum* L., 15% common vetch, *V. sativa* L., and 15% purple vetch, *V. benghalensis* L. By seed weight mustard included 61% white mustard, ‘IdaGold’ *Sinapis alba* L., and 39% India mustard, ‘Pacific Gold’ *Brassica juncea* Czern.

c During the first 6 years of the study, Systems 1 and 2 were fallow all winters except the winter of year 4. All other systems were cover cropped every winter.

d Seeding rates are referred to as 1× and 3×, where 3× is 3 times greater than 1×. The 1× and 3× rates in kg ha^-1^ were 11 and 33 for mustard, 90 and 270 for rye, and 140 and 420 for the legume-rye mixture.

e The compost was made from urban yard waste and the annual application (oven dry basis) was 15.2 Mg ha^-1^. It was applied in a split application annually with half before each of the two vegetable crops.

f Total, cumulative organic matter input (oven dry basis) from cover crop shoots + compost over the 6 years.

**Table 2:** Raw data of soil enzyme activities and microbial biomass in the beginning of the study, 6 years later, and the change over 6 years in the Salinas Organic Cropping Systems experiment in Salinas, California. This includes data from all eight systems in the experiment. The related articles in *Soil Biology and Biochemistry* (SSB) [1] and the *Soil Science Society of America Journal* (SSSJA) [2] only included data from five of the eight systems with optimal seeding rates for weed suppression. A Microsoft Excel version of the table in available in the supplementary material (Supplementary Table 1).

## 2 Experimental design, materials, and methods

The ongoing SOCS experiment began in 2003 and occurs in a 0.9 ha field that includes 32 plots, organized in 4 blocks of 8 system plots per block. The first eight years of this study were focused on vegetable production (lettuce followed by broccoli most years) in 8 systems that differed in compost inputs and cover crop (type, seeding rate and frequency) (Table 1). The annual rotation began in October or November each year and included either a winter fallow or winter cover crop that grew until February or March and was usually followed by the two vegetable crops. Winter weed growth in system 1 and 2 that were fallow most winters were managed with shallow tillage as needed, to minimize weed growth and prevent weed seed production, but otherwise, tillage was consistent across all systems. Other than the differences in cover crop and compost inputs between systems, all management (i.e., pest control, tillage, harvest schedules) and inputs (i.e., irrigation, fertilizers) were equivalent across all systems during the vegetables crops [3], [5], [1].

Soil samples for analysis of microbial biomass carbon and nitrogen, and enzyme activities were collected to a depth of 0 to 6.5 cm from 6 to 8 cores in each plot and were bulked and archived in a freezer at -25 °C until they were analyzed. Microbial biomass carbon and nitrogen were determined using the chloroform fumigation–extraction method [4], [9] and soil enzyme activities were determined using colorimetric and titration methods [8], [6], [7] as described in detail in our related articles [1], [2]. To evaluate changes in microbial biomass and enzyme activities over time, the analyses were done on soil collected at time 0 (October 2003 just prior to the application of the treatments) and after six years of management. The data presented here include the raw data for all eight systems in the experiment (Table 2), whereas the data for only five systems were used in the analyses in the related articles [1], [2]. Fig. 1, Fig. 2, Fig. 3, Fig. 4, Fig. 5, Fig. 6, Fig. 7, Fig. 8 illustrate major patterns in the data with the some of the raw data plotted with means and 95% confidence intervals. We refer readers to our most recent related article [2] for an explanation of how in compare systems using 95% confidence intervals in this study and how the ESCI software (available at https://thenewstatistics.com/itns/esci/) can help with these comparisons.
