# Supplementary material for: Soil microbial biomass and enzyme data after six years of cover crop and compost treatments in organic vegetable production
Source: Data Brief. 2018 Sep 12;21:212–27. doi: 10.1016/j.dib.2018.09.013 (PMC6197954; doi:10.1016/j.dib.2018.09.013)
Supplement: Supplementary file 1 — Supplementary material [file mmc1.docx]

Conflict of Interest and Authorship Conformation Form

Please check the following as appropriate:

✓ All authors have participated in (a) conception and design, or analysis and interpretation of the data; (b) drafting the article or revising it critically for important intellectual content; and (c) approval of the final version.

✓ This manuscript has not been submitted to, nor is under review at, another journal or other publishing venue.

✓ The authors have no affiliation with any organization with a direct or indirect financial interest in the subject matter discussed in the manuscript

- The following authors have affiliations with organizations with direct or indirect financial interest in the subject matter discussed in the manuscript:

Author’s name Affiliation
